# Breath Sensor Technology for the Use in Mechanical Lung Ventilation Equipment for Monitoring Critically Ill Patients

**DOI:** 10.3390/diagnostics12020430

**Published:** 2022-02-07

**Authors:** Manohar Prasad Bhandari, Viktors Veliks, Ilmārs Stonāns, Marta Padilla, Oļegs Šuba, Agija Svare, Inga Krupnova, Ņikita Ivanovs, Dina Bēma, Jan Mitrovics, Mārcis Leja

**Affiliations:** 1 Institute of Clinical and Preventive Medicine, University of Latvia, LV-1586 Riga, Latvia; viktors.veliks@lu.lv (V.V.); ilmars.stonans@lu.lv (I.S.); dina.bema@lu.lv (D.B.); 2 JLM Innovation GmbH, D-72070 Tubingen, Germany; marta.padilla@jlm-innovation.de (M.P.); jan.mitrovics@jlm-innovation.de (J.M.); 3 Riga East University Hospital, LV-1038 Riga, Latvia; olegs.suba@aslimnica.lv (O.Š.); agija.r@gmail.com (A.S.); inga.krupnova@aslimnica.lv (I.K.); nikita.ivanovs@gmail.com (Ņ.I.); 4 Faculty of Medicine, University of Latvia, LV-1586 Riga, Latvia

**Keywords:** VOC sensors, mechanical ventilation, exhaled breath, COVID-19, patient monitoring

## Abstract

Background: The need for mechanical lung ventilation is common in critically ill patients, either with COVID-19 infection or due to other causes. Monitoring of patients being ventilated is essential for timely and improved management. We here propose the use of a novel breath volatile organic compound sensor technology to be used in a mechanical lung ventilation machine for this purpose; the technology was evaluated in critically ill COVID-19 patients on mechanical lung ventilation. Methods: Based on the consistency results of our study data, the breath sensor device with metal oxide gas sensors and environment-controlling sensors was mounted on the ventilation exhaust port of the ventilation machine; this allowed to ensure additional safety since the device was placed outside the contour between the patient and equipment. Results: The sensors allowed stable registration of the signals for up to several weeks for 10 patients in total, depending on the storage amount; a proportion of patients were intubated or received tracheostoma during the evaluation period. Future studies are on the way to correlate sensor readings to other parameters characterizing the severity of the patient condition and outcome. Conclusions: We suppose that such technology will allow patient monitoring in real-time for timely identification of deterioration, potentially requiring some change of management. The obtained results are preliminary and further studies are needed to examine their clinical significance.

## 1. Introduction

Mechanical ventilation is a life-saving medical intervention in patients experiencing respiratory failure in the case of critical illness, including but not limited to COVID-19. These patients are at risk of multiple complications, and require intensive monitoring to identify signs of clinical worsening and to minimize the risk of iatrogenic harm [1]. Pulse oximetry, capnography, monitoring of driving pressure, transpulmonary pressure, and the pressure-volume loop, as well as adjustment of airway cuff pressures are being used in these patients; however, each of the methods has its limitations [1]. It is important to ensure reliable monitoring of critically ill patients, including those with COVID-19, during mechanical ventilation to minimize harm to the patients.

The rapid detection of COVID-19-specific breath volatile organic compounds (VOCs) would be a great step forward for diagnostic purposes and for monitoring disease progression or responses to conventional or investigational drugs. Here, we present a novel sensor-based VOC technology to be assembled within the artificial ventilation equipment for severely ill patients, including those with COVID-19. The major advantages of the VOCs sensor device combined with the mechanical ventilation system are its simple and robust design, non-invasiveness, easy operation, fast response, reusability, application in hospital usage, convenient data acquisition and data exchange, and low manufacturing costs.

The rapid spread and transmission of COVID-19 has threatened global health, leading to an urgent need of new technology for its monitoring and diagnosis. The analysis of VOCs in exhaled human breath has become a growing field of research in recent years because of the advances in analytical techniques and nanotechnology [2,3]. These compounds may provide valuable information about the metabolic activity of the enzymes or pathological processes of any disease. The VOCs profile associated with COVID-19 infection will provide information on potential prognostic features. Metal oxide (MOX) gas sensors are most frequently used in VOC testing approaches. They are usually cross-reactive and have high sensitivity to VOCs. Moreover, such sensors are sensitive under high humidity conditions, which is an important requirement for exhaled breath analysis. MOX-based chemiresistive sensors have been receiving attention due to their high potential for miniaturization of portable diagnostic tools. Sensor technology application for VOCs detection in human breath for disease monitoring is easy-to-use, non-invasive, fast, and low-cost [4,5,6]. An array of semiconducting MOX sensors along with methods of pattern recognition and analytics is capable of detecting a compendium of VOCs in exhaled breath. Each sensor responds to a range of VOCs, which allows the sensing and analysis of their pattern from a mixture of compounds. The isolation of disease-specific VOCs and their detection via breathomics and novel sensing materials highlights the clinical applicability of exhaled breath monitoring [7,8].

Few previous studies have addressed the use of VOCs in the monitoring of ventilated patients, suggesting this to be a valuable strategy for non-invasive control and optimization of ventilation strategies [9,10,11]. This has been further endorsed in studies involving COVID-19 patients by suggesting that particular breath chemistries (namely, methylpent-2-enal, 2,4-octadiene 1-chloroheptane, and nonanal) could be identified in the breath of these patients developing acute respiratory distress syndrome (ARDS), a condition that can be treated with mechanical ventilation [12]. Importantly, mechanical lung ventilation is associated with a significantly increased risk of hospital infection [13], which is also the case for COVID-19 patients [14]. A study involving the use of a novel breath detector device with a catalytically active, resistive chemosensor that is highly selective to NO and ammonia was able to detect the distinct breath signature of COVID-19 within 15 seconds [15]. A multiplexed nanomaterial-based hybrid sensor array utilizing advanced machine learning and feature extraction algorithms enabled the detection and monitoring of COVID-19 from exhaled breath [16]. This gave us a good reason to hypothesize that sensor-based VOC breath technology could become a reliable tool for monitoring critically ill patients, including those with COVID-19.

However, multiple challenges with the application of this technology for the abovementioned purposes do exist. Sensor readings could be substantially influenced by breath humidity [17], medications, clinical parameters, inter/intraperson variability of the VOC profile, and other external variables, such as patient condition, coughing, mucous, ventilation tube movements, and mechanical issues in the lung ventilation machine [18]. In addition, cross-sensitivities to environmental and external variables influencing the environmental sensors affect the gas sensors response, as well as sensor drift and sensor warming-up [19,20]. To counteract such unwanted effects, appropriate signal pre-processing can be applied and a multivariate predictive model is built using statistical and machine learning methods. These limitations are addressed by the proposed breath sensor technology, so that the effect of the evolution on the patients’ state and breath characteristics can be isolated and thus monitored.

## 2. Materials and Methods

We designed a sensor-based VOC technology and performed the initial validation of the technology in critically ill patients with COVID-19 infections being managed in one of the two departments of Riga East Clinical University Hospital–Clinic of Toxicology and Sepsis or Latvia Infectology Centre. The details of its design, the placement of the sensor device in the mechanical lung ventilation machine where it can be best used, sensor set, requirements that are met by this modular device in the clinical setup, data acquisition, and sensor reading results are explained.

### 2.1. Design and Engineering of the Sensor Device

The design of the technology was performed by JLM Innovation GmbH (Tübingen, Germany) based on the definition of the need defined by the clinical partners. The sensor device comprises the environmental sensors that measure temperature, pressure and humidity, and a set of MOX gas sensors with different specificities measuring VOC patterns present in the patient’s breath. The registration device is built on a Raspberry Pi platform and the data recording is achieved through a Python script.

**Figure 1:**
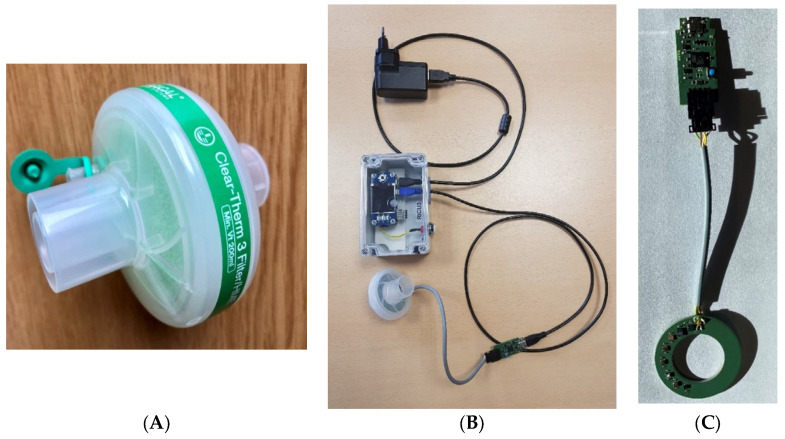
(**A**) Clear-Guard MIDI breathing filter with Luer port; (**B**) sensor device adapted for use in the mechanical lung ventilation machine; the device embedded in the Clear-Guard MIDI breathing filter, a sensor signal registration device (Raspberry Pi), and a power adapter; (**C**) sensor board connected to a micro-USB cable.

The gas sensors are housed in a Clear-Guard MIDI disposable filter housing with a Luer port that is connected to the mechanical lung ventilation machine. The filter with the sensors is disposable, while the sensor signal registration device, which is not in direct contact with the patient’s breath, is reusable. Figure 1A shows a model of a Clear-Guard MIDI breathing HME (Heat and Moisture Exchanger) filter (Intersurgical Ltd., Wokingham, UK), wherein the sensor device is integrated. A complete set-up of the sensor device adapted for use in the mechanical ventilation equipment is shown in Figure 1B. Figure 1C shows the sensor board connected to a micro-USB cable for the sensor signal recording. The device can be controlled and data acquisition could be done via the JLMlogSP software using a Wi-Fi transmitter. This would make the sensor device more efficient to be deployed at the hospital.

**Figure 2:**
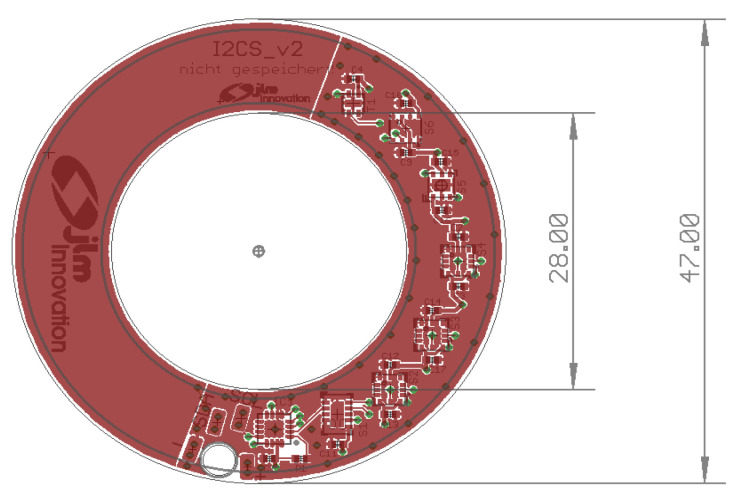
Technical design of the sensor device (all dimensions are in mm). The sensors are on the right side.

The technical design of the sensor device electronics is shown in Figure 2. All of the sensors are embedded on the right side of the device.

The sophisticated and unique use-case of breath VOC sensor technology having incorporated into the mechanical lung ventilation machine and meeting various specifications was tested and evaluated in real conditions on patients suffering from COVID-19.

### 2.2. Placement of the Sensor Device

**Figure 3:**
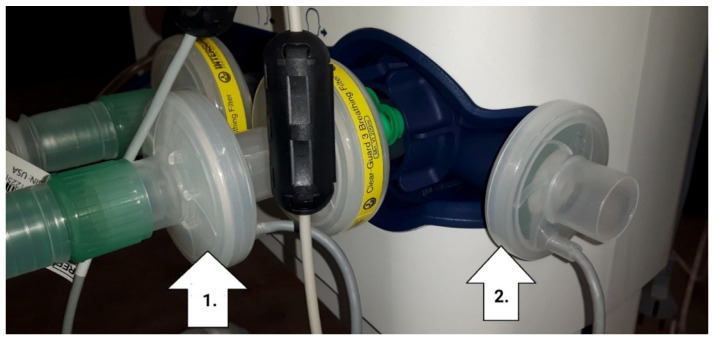
Sensor device placement at the outlet of the mechanical lung ventilation machine (position 2) in a real operating condition in a hospital setting, which was adopted. Another potential location (position 1) of the breath contour before the ventilation machine was also evaluated.

Our team of medical doctors, researchers, and engineers explored the concerns around the appropriate placement of the sensor device in the mechanical lung ventilation machine (HAMILTON-C6, Hamilton Medical AG, Bonaduz, Switzerland) to address the efficacy of the device. For this purpose, possible allocations of the sensor device in the ventilation machine and the stability of the MOX gas sensor readings at those locations were evaluated (Figure 3). An array of semiconducting MOX gas sensors and an environmental sensor module was integrated into the ventilation machine to help monitor patients’ state and recovery.

Taking into consideration the potential risk of contamination, safety, practicality, breath humidity, and sensor interaction with the infectious material, the outflow part of the ventilation machine was identified and selected to be the optimal place for sensor localization (on the right side of Figure 3, position 2). The installation of the sensor module on the ventilation exhaust port or outlet was carried out and the breathing measurement data were obtained, hence ensuring the normal operation of the mechanical ventilator in safety conditions for the patients and the clinicians. Since the sensor readings were stable, the outlet of the ventilation machine was chosen to exclude any contact to the airflow between the patient and the ventilator. The humidity was also measured, and it did not have any impact on the chosen position. There, the sensor device does not come into contact with the patient, does not interfere with the treatment, and is less exposed to the adverse conditions.

**Figure 4:**
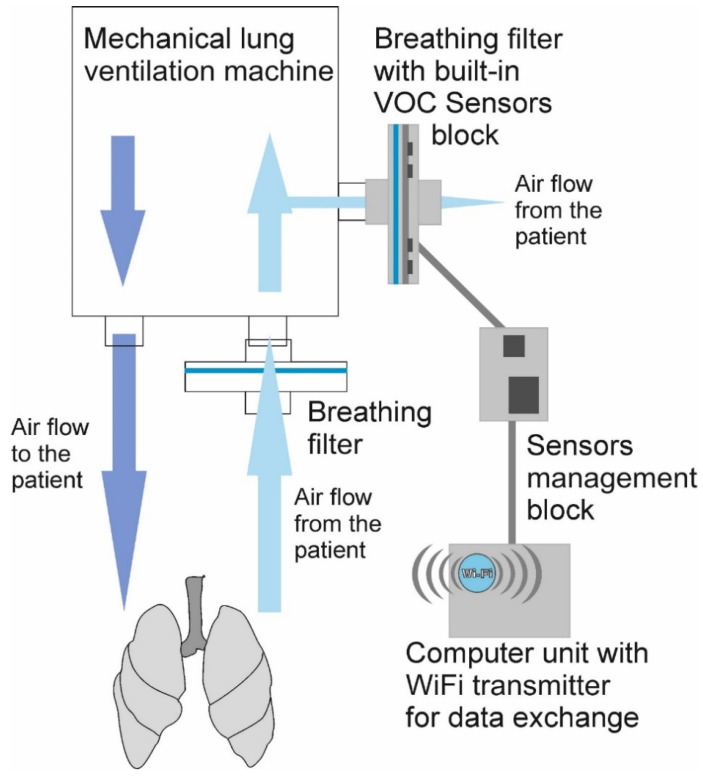
Schematic of the VOC sensor device placement including the ventilation airways and sensors’ signal acquisition for the monitoring of COVID-19 patients.

Figure 4 shows a schematic representation of the whole system including the ventilation airways and modules, sensor placement, sensors signal recordings, and data exchange using a Wi-Fi transmitter. The construction and localization of the breath sensor device is designed to enable the use of the instrumentation for monitoring severely ill patients connected to the mechanical lung ventilation machine. From the patient, the expiration air flows through the intubation tubes and relevant bacterial filters into the VOC sensor block located at the exhaust port of the mechanical lung ventilation machine. The airflow from the sensor system does not return to the patient. The breathing measurement data from the sensor readings are recorded using a data cable and a Wi-Fi transmitter for further processing and analysis. The Wi-Fi transmitter works only while performing the data exchange and it is turned off during the observation. This is important for the safety of other medical devices. The sensor device can be connected to a Windows PC and controlled via the in-house JLMlogSP software. The output data are generated in a text file format, which can be processed using multivariate signal processing for further analysis and visualizations.

### 2.3. Sensors Characteristics

The sensor device is equipped with environmental sensors that measure temperature, pressure, and relative humidity, and an array of chemiresistive MOX gas sensors. Table 1 summarizes the description of the sensors used in the breath sensor module together with their main features. The gas sensor array contains six digital gas sensors: one sensor with two outputs for VOC sensing (SGP30), two sensors with one output for VOC sensing (CCS811 and BME680), and three sensors each modulated at three temperature levels through heater voltage regulation (IDT-ZMOD). Modulation in temperature is an interesting property of the MOX sensors, which all need to be heated so that the chemical reactions can take place at their sensing layer. MOX sensors show different sensitivities when heated at different temperatures. For this reason, having three sensors (IDT-ZMOD) heated at three different temperatures is equivalent to having nine sensors. Therefore, the array gives 13 sensor signals which provide a specific fingerprint on the VOC content in the exhaled human breath. All MOX gas sensors have broad sensitivity to the VOCs in the breath, responding to the presence of many types of VOCs produced by internal biochemical processes of the human metabolism [21].

These sets of sensors were selected for the designated task based on sensing characteristics, low power consumption, technological quality, low cost, and compatibility with medical applications. The sensor module was connected to the exhaust port of the mechanical lung ventilation machine and allowed continuous measurements of exhaled breath and acquisition of signals every second during the non-invasive ventilation of COVID-19 patients.

## 3. Results

### 3.1. Sensor Signal Recording

After the approval of the sensor device placement in the mechanical lung ventilation machine, sensor placement in a hospital setting was started. The device was tested on patients with severe COVID-19 infection under treatment. The data were collected by small Raspberry Pi, which later transmitted the data via a Wi-Fi transmitter. The breathing measurement data from the patients were recorded which could be used to monitor changes in the VOCs present in the exhaled breath of the patients connected to the ventilation machine. The presence of endogenous VOCs in exhaled breath gives knowledge about their pathophysiological origins and the biochemical pathways involved in disease development. They serve as preclinical biomarkers of various diseases [22].

**Figure 5:**
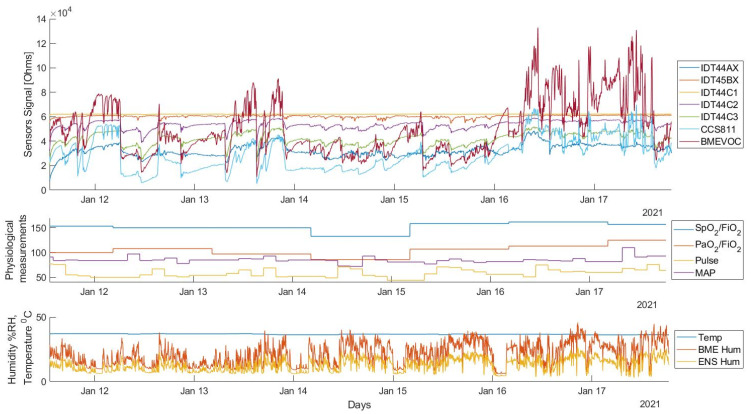
Sensor signals obtained during the ventilation of a patient (total recording time or full registration period). The measurements refer to one-week period.

Figure 5 reports an example of signals acquired from all the sensors during the ventilation of a COVID-19 patient in a real operating condition for one week, with additional external measurement information on the ratio of peripheral arterial oxygen saturation to the fraction of inspired oxygen (SpO_2_/FiO_2_), the ratio of partial pressure arterial oxygen to the fraction of inspired oxygen (PaO_2_/FiO_2_), the pulse, and the mean arterial pressure (MAP) data. The acquired raw sensor signals in resistance were normalized to compare the sensor responses. It can be observed that all the gas sensors, each with its own response time, showed a good correlation in different conditions of the patient, with the ability to catch every single respiratory act. All sensors were stable during the long-time measurement period and delivered good resolution readings; even before the sensors became stable, the sensor response signals could be identified. By comparing the temperature, humidity, and pressure sensor data with the gas sensor data, it can be seen that the gas sensors pick up small independent signals.

The signals from the MOX gas sensors vary during the ventilation period to exhaled VOC content indicating a promising sensitivity to exhaled VOCs. Multivariate signal processing of the sensors array data will help to predict the condition/course of the disease, correlating the obtained sensor data with the clinical information, which will help the doctors adjust their treatment, for example by adding medicines with potential antiviral activity. At the same time, the sensor measurement data could help diagnose the potential nosocomial infections and antibacterial management in a timely manner. It is necessary to filter out the indicators responding to the change of the medical intervention of the patient that affects the sensors’ response.

**Figure 6:**
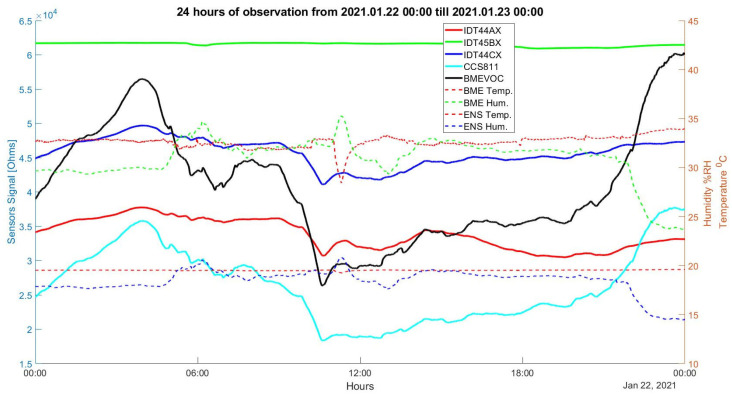
Environmental BME and ENS sensors, and gas sensor signals taken from a patient in the mechanical lung ventilation machine for 24 h of measurements.

Figure 6 shows a segment of the recorded sensors’ signals towards environmental sensor data for one patient during 24 h of observation. Environmental sensors record temperature and humidity changes in the breath of the patient and are measured by two sensors: the first one is an ENS210 located separately on the board, and the second is a part of BME680 sensors. The temperature sensor in the BME680 is affected by the sensor heating, and for this reason, an external sensor ENS210 is included in the device. Both humidity sensors show similar patterns of humidity changes.

### 3.2. Data Exploration and Methodology

In this section, we explore the data collected from eight patients using Principal Component Analysis (PCA) which allows for the visual inspection of the data structure on a new space of smaller dimension. In such a space, the orthogonal axes or Principal Components (PCs) are such that they capture sequentially decreasing amounts of data variance. Therefore, PC1 captures more variance than PC2, and so on. A PCA scores plot represents the data projected in the first two PCs of this PCA space (PC1 and PC2), which are thus the two PCs that capture the data variability the most. Indeed, PCA is a convenient way to represent multivariate data, as the PCs are linear combinations of the original sensors.

The dataset consists of sensor measurements from the breath of 10 patients across a few days. Data from two of the patients were discarded due to the short measurement time or bad data quality because of individual sensors’ malfunction. Table 2 shows basic information about the patients. Notice the variability in the samples; the patient set includes both genders with ages within a 25-year range (51–76), BMI between 23 and 45, two clinical sites, intubated or not, two final conditions (deceased or survived), four different devices, and a variable number of days of measurements.

Before the PCA computation, we performed a few pre-processing steps. First of all, we removed two of the IDT-ZMODA sensors due to an extremely high correlation among them. In addition, we removed the first three hours of measurements because the sensors were showing large transients due to not being warm enough for stable responses. Other performed tasks, such as reducing the correlation to environmental variables, are detailed in the Supplementary Material.

**Figure 7:**
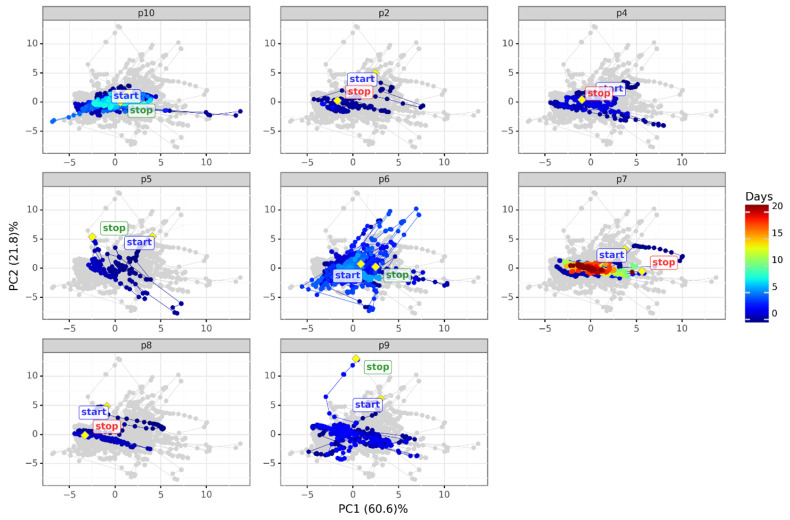
PCA scores plot per patient across time, capturing 95% of the variation of the patients’ data in the first two days of measurements. The color of the STOP label indicates whether the patient survived (green) or died (red). The grey shadow indicates the silhouette of all the data in PC1 vs. PC2.

**Figure 8:**
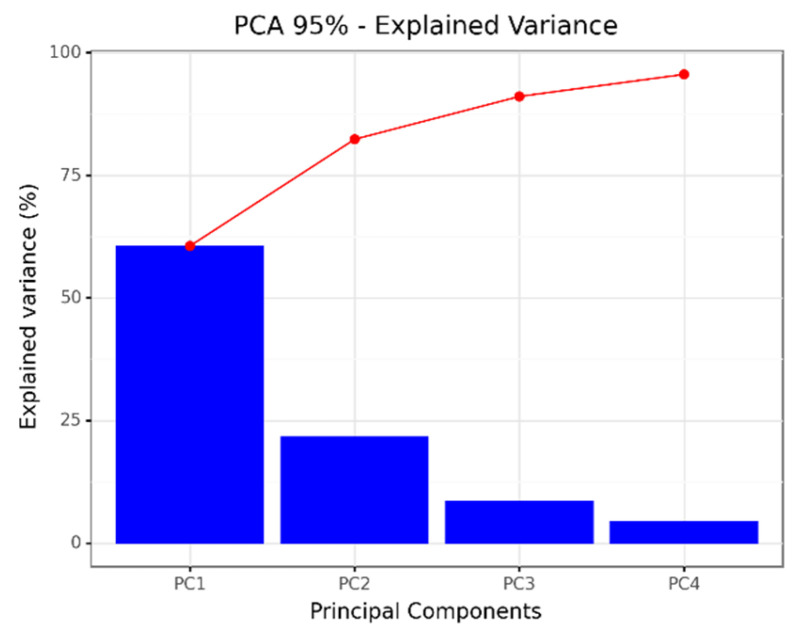
PCA explained variance plot.

**Figure 9:**
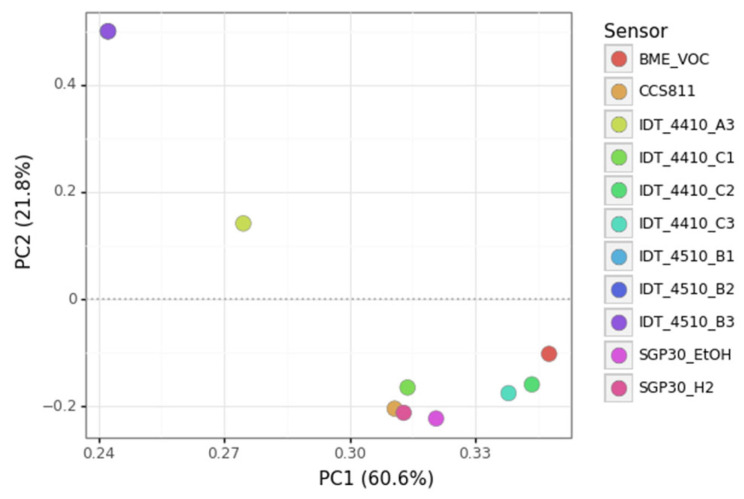
PCA loadings plot.

After the above-mentioned pre-processing steps, because we only had data from eight patients, we built a PCA model with the complete data set. Figure 7 shows the scores plot of a PCA model capturing 95% of the variability of all patients’ data from the first two days of measurements, resulting in four principal components (PCs), as shown in Figure 8. The loadings plot in Figure 9 shows the contribution of the sensors to the first two PCs. Unfortunately, we have quite different amounts of data for different patients, and for this reason, we chose to build the PCA model with the first two days of measurements of all the patients and visualize the evolution of a few patients (p6, p7 and p10) with respect to the model.

For better visualization of the PCA scores plot (Figure 7), the figure shows in grey the silhouette of the whole scores data, and then highlights the scores corresponding to each patient in separated panels with the patient number on top. The scores point colors correspond to the measurement time. Two large yellow points with a diamond shape and their corresponding label indicates the start (START) and the end (STOP) of the measurements. The color of the STOP label indicates the final status of the patient: survived (green) or deceased (red).

## 4. Discussion

A lot of effort is being made to get technological advances in developing novel analytical and sensing platforms for monitoring critically ill patients, including those with COVID-19. In this context, the scientific community believes that the exhaled breath analysis will realize its long-held potential becoming a revolutionary tool in personalized medicine and daily clinical practice [22,23]. Nano gas sensor-based approaches are effective in non-invasive diagnosis and the detection of the coronavirus because of their high detection capability, stability, reliability, design, and low cost [24,25]. The potential and different alternatives have been evaluated for the use of breath sensor technology in monitoring critically ill and COVID-19 patients. In fact, different types of nanomaterials have attracted the attention of the scientists in diagnosis, detection, monitoring, and therapy applications against COVID-19 [26]. Looking at the pathophysiology of COVID-19, there are several indicators that a coronavirus infection is detectable in the breath VOC pattern. The disease has been reported to cause a multitude of symptoms and can affect several organs. This supports the assumption that the metabolism is affected in multiple ways and that the volatile metabolite distribution is altered. Thus, a spectrum of VOCs could be used for monitoring the disease progression and effect of a therapeutic experimental drug, which would speed up the development of an efficient treatment for COVID-19.

We have designed and developed a novel breath analysis system based on gas sensor technology for monitoring severely ill COVID-19 patients in the mechanical lung ventilation machine. The sensor module is thought to enhance the monitoring features of the mechanical lung ventilation machine at hospital, providing environmental and gas sensors to analyze the exhaled breath of COVID-19 patients. The decision to connect the sensor system at the outlet of the ventilation equipment was approved based on the patient safety, efficacy of the sensor device, analysis of the breathing measurement data, and different considerations.

To test the performance of the new breath analysis system, we collected measurements from a set of eight patients (2 of the 10 patients discarded) during a variable period of time (from around 2 to 20 effective days). After few pre-processing steps to prepare the data (down-sampling, filtering, and decreasing the correlation with environmental variables), to explore the structure of the data we built a PCA model in the first two days of measurements for all the patients, retaining 95% of the variance in four features (Figure 8). The loadings plot (Figure 9) shows a high degree of correlation among sensors of similar type: IDT-ZMODB, SGP30, where some seem not to be present in the plot because the points are superimposed. Sensor redundancy is useful in sensor arrays for fault detection and correction, but this is not a priority in this application. In future system developments, we will consider alternative sensor technologies.

The PCA scores plot in Figure 7 shows the evolution of the condition of the patients in the first two PCs. Data for most patients correspond to the training set (dark blue for the first 2 days of measurements) and contain large variations that may correspond to changes in the patient status due to medicine intake, rotation in the patient’s position, eating, or other episodes. We can see how the evolution of the patient’s condition drives the ‘stop’ point far from the ‘start’ point (patients p9, p7, for example) or the contrary (p10, p6) in only two principal components. Data from several more patients are needed to find patterns that can help predict the patient’s next status, such as worsening of his/her condition, so that the clinicians can act in advance. Such patterns would be based on breath analysis data, such as the one given by the system described here, or in combination with other parameters automatically collected from the patient.

From a technical point of view, many mechanical lung ventilators are available with different levels of complexity and sophistication. Because of the overwhelming health emergency caused due to COVID-19, the application of different rules in healthcare provision, including indications for artificial lung ventilation, is warranted [27]. This work reported on assessing the feasibility of implementing exhaled breath analysis through real-time sensor monitoring for critically ill COVID-19 patients in artificial ventilation equipment. The system followed the patient’s state in real-time that could help to predict the risk of possible complications by studying the sensor signals. Therefore, it is possible to safely incorporate the developed breath sensor technology into the mechanical lung ventilation machine, and it has the potential to monitor critically ill patients, including those with COVID-19.

The urgency of the COVID-19 pandemic necessitates the use of such an innovative technology that can be used to forecast disease progression, to alert clinicians about the changes in patients’ health, and to adjust their treatment. It is expected that, as the patient’s sickness evolves, the VOC content in his/her breath changes. The study of the sensors signals monitoring the VOC pattern in the patient’s breath during several days will allow to define and predict the patient’s evolution in COVID-19, which in turn will help doctors to adjust therapy accordingly.

## 5. Conclusions

The preliminary results obtained are very promising and affirm that the developed breath sensor technology has potential in monitoring the course of severely ill patients, including COVID-19 patients, undergoing artificial ventilation. Furthermore, it is believed that the monitoring of the patients with severe COVID-19 infection with the sensor technology that is installed in the lung ventilation machine helps predict the course of the disease, and that it selects the subjects that are going to develop more severe complications. This will facilitate the decision-making on the management and treatment of severely ill COVID-19 patients. The sensor technology could be implemented for clinical use in the hospitals, therefore in the interests of the severely ill COVID-19 patients. This application may have wider implications beyond COVID-19 patients.

In summary, the initial clinical experiments and the data obtained so far in the patients with severe COVID-19 infection provide the proof and solution for the applicability of this sensor technology in these settings for long-term patient monitoring. In future work, the clinical data and other parameters will be registered and correlated to the sensor readings to evaluate the severity of the disease.

## Figures and Tables

**Table 1:** Description of the sensors used in the sensor device.

Manufacturer	Sensor Type/Model	Technology	Number/Subtype	Sensor Main Feature
Renesas/IDT	ZMOD4410A	MOX	3	One output for VOCs at three different temperatures
Renesas/IDT	ZMOD4410C	MOX	3	One output for VOCs at three different temperatures
Renesas/IDT	ZMOD4510B	MOX	3	One output for VOCs at three different temperatures
AMS/ScioSense	CCS811	MOX	1	One output for VOCs
Sensirion	SGP30	MOX	2	Two outputs for EtOh and for H_2_
BOSCH	BME680	MOX	1	One output for VOCs, three outputs for environmental variables: temperature, pressure, and humidity
AMS/ScioSense	ENS210	-	2	Two outputs for temperature and humidity

**Table 2:** Information about the patients.

Patient Number	2	3	4	5	6	7	8	9	10
Age	67	71	61	64	55	54	54	76	51
Sex	F	M	M	F	M	M	F	M	F
BMI	35.2	24.7	23.1	23.5	30.86	30.9	37.2	38.7	44.6
Noninvasive Ventilation	No	No	Yes	Yes	Yes	Yes	Yes	Yes	Yes
Intubation	Yes	Yes	Yes	No	No	Yes	Yes	No	No
Deceased/Survived	D	D	D	S	S	D	D	S	S
Clinical Site	G ^1^	G ^1^	G ^1^	G ^1^	LIC ^2^	G ^1^	LIC ^2^	G ^1^	G ^1^
Start Date	9 December 2020	10 December 2020	17 December 2020	29 December 2020	11 January 2021	9 January 2021	22 February 2021	24 February 2021	26 March 2021
Stop Date	16 December 2020	16 December 2020	23 December 2020	31 December 2020	17 January 2021	30 January 2021	25 February 2021	27 February 2021	3 April 2021
Number of Days	8	7	7	3	7	21	4	4	8

^1^ Gaiļezers Hospital; ^2^ Latvia Infectology Centre.

## Data Availability

The data supporting the reported results are available on request from the corresponding authors.

## Supplementary Materials

The following supporting information can be downloaded at: https://www.mdpi.com/article/10.3390/diagnostics12020430/s1, File S1.
