# Supplementary material for: Breath Sensor Technology for the Use in Mechanical Lung Ventilation Equipment for Monitoring Critically Ill Patients
Source: Diagnostics (Basel). 2022 Feb 7;12(2):430. doi: 10.3390/diagnostics12020430 (PMC8870831; doi:10.3390/diagnostics12020430)
Supplement: Supplementary file 1 [file diagnostics-12-00430-s001.zip › diagnostics-1557110-supplementary.pdf]

Pre-processing steps of the gas sensors signals

Before the PCA computation, we performed few pre-processing steps. First of all, we removed two of the IDT-A sensors due to extreme high correlation among them. In addition, we also removed the first 3 hours of measurements because the sensors were showing large transients due to not being warm enough for stable responses. Then, we down sampled the dataset (including the environmental sensors data) to one data point every 10 minutes calculated as the median of the data points within that 10 minutes of time. And finally, a median filter was applied with a small window of 5 points to slightly smooth the signal.

The gas sensors pre-processing tasks consisted of two steps. First, for every patient and every sensor, we considered the first measurement point as a measure of the sensor baseline (R_o) and computed the ratio with the remaining data (R) as R_o/R. Then, we reduced the correlation between the sensors signals and the environmental data (temperature and humidity from BME sensors) using Partial Least Squares (PLS). For the PLS procedure, we took data subsets of about 200 data points per sensor and computed a PLS model with one component between the environmental data X_{env} and each sensor y_i as:

$$y_i = X_{env}^T \cdot b_i + e_i$$

Where b_i is the regression vector and e_i is the error. The PLS model results in a predicted variable y that is most correlated with the data X, thus, to remove much of the correlation between X_{env} and y_i, we subtract the predicted sensor signal $\hat{y}_i$ in eq. 1 to the original sensor signal y_i:

$$\hat{y}_i = X_{env}^T \cdot b_i \qquad (1)$$

$$y_i' = y_i - \hat{y}_i$$

The result of this PLS-based method for reducing the correlation between the environmental variables and the sensor signals is a reduction of the average correlation (in absolute values) from 36±12% to 7±4%.
